# Synergistic inhibition of human cytomegalovirus replication by interferon-alpha/beta and interferon-gamma

**DOI:** 10.1186/1743-422X-2-14

**Published:** 2005-02-23

**Authors:** Bruno Sainz, Heather L LaMarca, Robert F Garry, Cindy A Morris

**Affiliations:** 1 Department of Microbiology and Immunology, Program in Molecular Pathogenesis and Immunity, Tulane University Health Sciences Center, 1430 Tulane Avenue, SL-38, New Orleans, LA, 70112, USA

## Abstract

**Background:**

Recent studies have shown that gamma interferon (IFN-γ) synergizes with the innate IFNs (IFN-α and IFN-β) to inhibit herpes simplex virus type 1 (HSV-1) replication *in vitro*. To determine whether this phenomenon is shared by other herpesviruses, we investigated the effects of IFNs on human cytomegalovirus (HCMV) replication.

**Results:**

We have found that as with HSV-1, IFN-γ synergizes with the innate IFNs (IFN-α/β) to potently inhibit HCMV replication *in vitro*. While pre-treatment of human foreskin fibroblasts (HFFs) with IFN-α, IFN-β or IFN-γ alone inhibited HCMV plaque formation by ~30 to 40-fold, treatment with IFN-α and IFN-γ or IFN-β and IFN-γ inhibited HCMV plaque formation by 163- and 662-fold, respectively. The generation of isobole plots verified that the observed inhibition of HCMV plaque formation and replication in HFFs by IFN-α/β and IFN-γ was a synergistic interaction. Additionally, real-time PCR analyses of the HCMV immediate early (IE) genes (IE1 and IE2) revealed that IE mRNA expression was profoundly decreased in cells stimulated with IFN-α/β and IFN-γ (~5-11-fold) as compared to vehicle-treated cells. Furthermore, decreased IE mRNA expression was accompanied by a decrease in IE protein expression, as demonstrated by western blotting and immunofluorescence.

**Conclusion:**

These findings suggest that IFN-α/β and IFN-γ synergistically inhibit HCMV replication through a mechanism that may involve the regulation of IE gene expression. We hypothesize that IFN-γ produced by activated cells of the adaptive immune response may potentially synergize with endogenous type I IFNs to inhibit HCMV dissemination *in vivo*.

## Background

Human cytomegalovirus (HCMV) is a ubiquitous beta-herpesvirus that affects 60–80% of the human population [1]. The lytic replication cycle of HCMV is a temporally regulated cascade of events that is initiated when the virus binds to host cell receptors. Upon entry into the cell, the viral DNA translocates to the nucleus, where expression of viral immediate early (IE), early and late genes occurs in a stepwise fashion [2]. While generally asymptomatic in immunocompetent individuals, primary HCMV infection may cause infectious mononucleosis and has been associated with atherosclerosis and coronary restenosis [3,4]. Furthermore, HCMV is the leading contributor of congenital viral infections in the United States and Europe, causing cytomegalic inclusion disease, pneumonia and severe neurological anomalies in infected neonates [5-7].

Like other herpesviruses, HCMV establishes lifelong latency in its host from which reactivation can occur and cause severe and fatal disease in immunocompromised individuals [8]. Cellular immune responses (MHC class I-restricted T-cells and natural killer (NK) cells) appear to be an important factor in both the control of acute infections and the establishment and maintenance of viral latency in the host [9-14]; however, the mechanisms by which T-cells affect HCMV replication are currently undefined. While cytotoxic T-cell activity has been shown to correlate with recovery from HCMV infection in patients [15,16], recent studies suggest that immune cytokines such as tumor necrosis factor-α and interferons (IFNs) may have direct inhibitory effects on HCMV replication [17,18]. In particular, the involvement of IFNs as a means of curtailing viral replication without cellular elimination is consistent with the hypothesis that cytokines produced by activated immune cells play a direct role in the control of viral infections [19-21].

Type I IFNs (IFN-α and IFN-β) and type II IFN (IFN-γ) are important components of the host immune response to viral infections. IFN-α and IFN-β are produced by most cells as a direct response to viral infection [22-24], while IFN-γ is synthesized almost exclusively by activated NK cells and activated T-cells in response to virus-infected cells [25]. Both types of IFNs achieve their antiviral effects by binding to their respective receptors (IFN-α/β or IFN-γ receptors), resulting in the activation of distinct but related Janus kinase/signal transducer and activator of transcription (Jak/STAT) pathways. The result is the transcriptional activation of IFN target genes and the synthesis of a number of proteins that interfere with viral replication (reviewed in [26]). Although IFNs are effective inhibitors of viruses such as vesicular stomatitis virus and encephalomyocarditis virus [26], almost all RNA and DNA viruses have evolved mechanisms to subvert the host IFN response [21,26,27]. For example, HCMV inhibits IFN-stimulated antiviral and immunoregulatory responses at multiple steps [24,28-32]. Likewise, the herpes simplex virus (HSV-1) protein ICP34.5 [33], the influenza A virus NS1 protein [34], the simian virus-5 V protein [35], the Sendai virus C protein [36], the hepatitis C virus (HCV) NS5A and E2 proteins [37] and the Ebola virus VP35 protein [38] have all been shown to block IFN-mediated responses in infected cells. However, several studies have shown that viruses normally resistant to the effects of type I or type II IFNs separately, are susceptible to IFNs when used in combination. For example, IFN-α/β and IFN-γ synergistically inhibit the replication of HSV-1 both *in vitro *and *in vivo *[20]. In addition, recent reports have indicated that IFNs used in combination have a synergistic antiviral activity against severe acute respiratory syndrome-associated coronavirus (SARS-CoV) [39], HCV [40] and Lassa virus [41].

In the present study, we examined the effects of IFN-α, IFN-β and/or IFN-γ on HCMV replication in human foreskin fibroblasts (HFFs). Treatment of HFFs with IFN-α, IFN-β or IFN-γ separately inhibited HCMV replication by ≤ 40-fold in both plaque reduction and viral growth assays. In contrast, treatment with IFN-α and IFN-γ or IFN-β and IFN-γ inhibited HCMV replication 10–20 times greater than that achieved by each IFN separately. This effect was synergistic in nature and the mechanism of inhibition may involve, at least in part, the regulation of IE gene expression. As with HSV-1 [20], we have found that when used in combination, both type I and type II IFNs potently inhibit the replication of HCMV *in vitro*.

## Results

### IFN-α/β and IFN-γ synergistically inhibit HCMV plaque formation

The abilities of human IFN-α, IFN-β or IFN-γ to inhibit the replication of HCMV were initially compared in a plaque reduction assay on HFFs. Viral plaque formation was reduced by 9-, 37- or 29-fold in fibroblasts treated with 100 IU/ml of IFN-α, IFN-β or IFN-γ, respectively (Table 1). To test the effects of combination IFN-treatments on viral plaque formation, HFFs were pre-treated with 100 IU/ml each of (1) IFN-α and IFN-β, (2) IFN-α and IFN-γ or (3) IFN-β and IFN-γ. As expected, the level of inhibition achieved with both IFN-α and IFN-β was not greater than the level of inhibition achieved by both IFNs separately. In contrast, pre-treatment with both type I IFNs (IFN-α or IFN-β) and type II IFN (IFN-γ) reduced HCMV plaquing efficiency by 164- and 662-fold, respectively (Table 1). To eliminate the possibility that this effect was merely a result of doubling the total amount of IFNs per culture, we tested the inhibitory effects of 200 IU/ml of each IFN separately. Two-hundred IU/ml of IFN-α, IFN-β or IFN-γ reduced HCMV plaque formation by only 11-, 37- or 30-fold, respectively (Table 1). The level of inhibition was not significantly greater than the level of inhibition achieved by each IFN at concentrations of 100 IU/ml (P > 0.05), suggesting that the degree of inhibition observed can be attributed to the presence of two distinct types of IFNs.

**Table 1:** Effect of IFN-α, IFN-β and/or IFN-γ on HCMV plaque formation

Treatment	IU/ml^a^	Log (mean no. of plaques) ± sem	Fold-inhibition^c^
Vehicle	---	3.34 ± 0.02^b^	---
IFN-α	100	2.38 ± 0.01*	9
IFN-α	200	2.30 ± 0.01*	11
IFN-β	100	1.77 ± 0.05*	37
IFN-β	200	1.77 ± 0.02*	37
IFN-γ	100	1.88 ± 0.03*	29
IFN-γ	200	1.85 ± 0.02*	30
IFN-α and IFN-β	100	1.95 ± 0.04*	25
IFN-α and IFN-γ	100	1.13 ± 0.09*	164
IFN-β and IFN-γ	100	0.52 ± 0.05*	662
IFN-α, IFN-β and IFN-γ	100	0.66 ± 0.15*	512

^a^HFFs were treated with either 100 or 200 IU/ml each of IFN-α, IFN-β or IFN-γ (separately or in combination).

^b^Mean ± sem of viral plaque formation on HFFs observed in 3 replicates per group. Cultures were infected with 2000 PFU/well of Towne-GFP, and plaque numbers were determined 14 d p.i. by fluorescent microscopy.

^c^Fold-inhibition was calculated as: 10^([log plaques / PFU in vehicle-treated] - [log plaques / PFU in IFN-treated])^

* Significant reduction in plaque numbers of IFN-treated groups as compared to vehicle-treated groups is denoted by a single asterisk (P < 0.001, one-way ANOVA and Tukey's post hoc *t *test).

**Figure 1:**
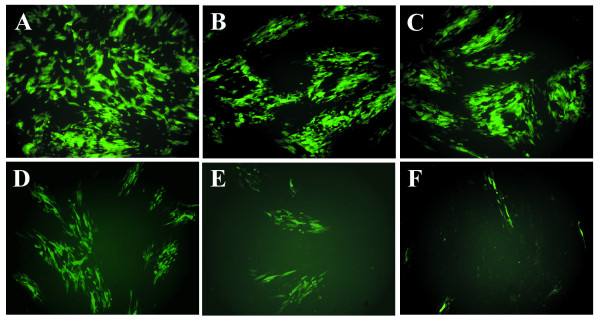
IFN-α, IFN-β and/or IFN-γ inhibit HCMV plaque formation on HFFs. HFFs were pre-treated with (A) vehicle or 100 IU/ml each of (B) IFN-α, (C) IFN-β, (D) IFN-γ, (E) IFN-α and IFN-γ or (F) IFN-β and IFN-γ. Monolayers were subsequently infected with 1000 PFU of HCMV strain Towne-GFP, and plaque numbers were determined 11 d p.i. by fluorescence microscopy. Plaques were determined by counting a minimum of 10 GFP-positive cells in one foci.

Figure 1 shows a representative micrograph of HCMV plaque formation on IFN-treated HFFs. Consistent with the results in Table 1, HCMV plaque efficiency was reduced and plaque morphology was smaller in cultures treated with a combination of type I and type II IFNs (Figure 1E, F). This phenotype was also observed in cultures treated with IFN-γ alone (Figure 1D), although the overall inhibitory effect of IFN-γ was similar to that achieved in IFN-β-treated HFFs.

**Figure 2:**
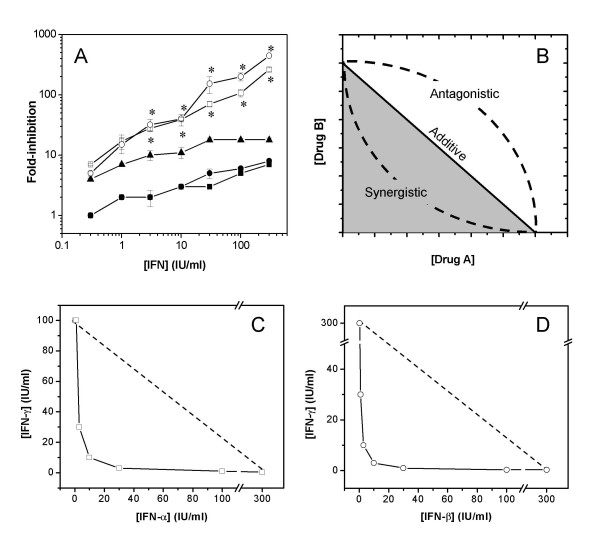
Type I IFNs (IFN-α and IFN-β) and type II IFN (IFN-γ) synergistically inhibit HCMV plaque formation on HFFs. (A) Viral plaque reduction assay. HFFs were treated with vehicle or increasing amounts of IFN-α (■), IFN-β (●), IFN-γ (▲), IFN-α and IFN-γ (□) or IFN-β and IFN-γ (○) prior to infection with 400 PFU of Towne-GFP (n = 3). Fold-inhibition in IFN-treated groups as compared to vehicle-treated groups is plotted as a function of IFN concentration (IU/ml). Significant differences in fold-inhibition for HFFs treated with combination IFNs relative to cells treated with individual IFNs are denoted by a single asterisk (P < 0.001, one-way ANOVA and Tukey's post hoc *t *test). (B) Illustration of a representative isobologram for a combination of two drugs. The solid line is the line of additivity. When the isobole lies below the line of additivity, the combinatorial effect of drug A and drug B is synergistic. When the isobole lies above the line of additivity, the combinatorial effect of drug A and drug B is antagonistic. Combination effect of (C) IFN-α and IFN-γ and (D) IFN-β and IFN-γ on HCMV plaque formation on HFFs was plotted in an isobologram. Values used to generate the concave isoboles were derived from a dose response curve and represent a combination dose required to elicit 95% (IC_95_) inhibition of viral plaque formation on HFFs. The dashed line represents the theoretical line of additivity.

The antiviral activity of IFNs on HCMV plaque formation was further assessed by generating dose-response curves (Figure 2A). The level of inhibition achieved with individual IFN treatments was ≤ 8-fold for IFN-α or IFN-β and ≤ 18-fold for IFN-γ at all concentrations tested. In contrast, combination IFN treatments achieved levels of inhibition 2–18 times greater than the sum of each individual IFN treatment. To determine if the enhanced inhibition of HCMV observed in HFFs treated with both type I and type II IFNs was synergistic, we employed the synergistic analysis for the determination of the interaction of two drugs [42,43]. Interaction indexes were initially calculated from the data generated in the dose response experiments (Figure 2A) to assess the synergistic potential of type I and type II IFN treatment. An interaction index of 0.05 ± 0.03 for IFN-α and IFN-γ combined and 0.04 ± 0.01 for IFN-β and IFN-γ combined indicated a high degree of synergy (Table 2). Additionally, synergy was confirmed by generating isobolograms in which concave isoboles are indicative of synergy while convex isoboles are indicative of an antagonistic effect (Figure 2B). Inhibitory concentrations were determined from dose response experiments, and IC_95 _isoboles were generated for HFFs treated with both IFN-α and IFN-γ (Figure 2C, concave plot) and HFFs treated with both IFN-β and IFN-γ (Figure 2D, concave plot). Consistent with the interaction indexes determined (Table 2), concave isoboles shown in Figures 1C and 1D indicate a synergistic relationship between type I IFNs (IFN-α and IFN-β) and type II IFN (IFN-γ), suggesting action via distinct antiviral pathways.

**Table 2:** Degree of antiviral interaction between IFN-α/β and IFN-γ

IFN Treatment^a ^(d_a _+ d_b_)	IC_90 _D_a_^b^	IC_90 _D_b_^b^	interaction index^c^
IFN-α + IFN-γ	300 IU/ml	30 IU/ml	0.05 ± .03
IFN-β + IFN-γ	100 IU/ml	30 IU/ml	0.04 ± .01

^a^HFFs were treated 12 h prior to infection with various combinations of type 1 IFNs (IFN-α or IFN-β) and type II IFN (IFN-γ).

^b^D_a _and D_b _are the concentrations of each IFN separately that inhibit HCMV plaque formation on HFFs by 90% (IC_90_).

^c ^Interaction index is a measure of the divergence between the amounts of IFNs that are observed to produce an inhibitory effect in combination (d_a _+ d_b_) and the amounts that would achieve the same effect separately (D_a _and D_b_). Indexes less than 1 indicate synergy, indexes greater than 1 indicate antagonism and indexes equal to 1 indicate additivity.

### IFN-α/β and IFN-γ synergistically inhibit HCMV replication

**Figure 3:**
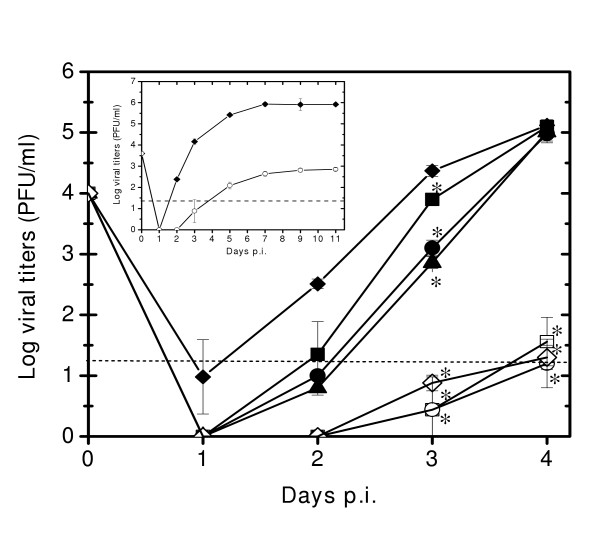
IFN-α, IFN-β and/or IFN-γ inhibit HCMV replication in HFFs. HFFs were treated with vehicle or 100 IU/ml of IFNs 12 h prior to infection with HCMV at a MOI of 2.5: (◆) vehicle, (■) IFN-α, (●) IFN-β, (▲) IFN-γ, (□) IFN-α and IFN-γ, (○) IFN-β and IFN-γ or (◇) GCV (100 μM). On the indicated d p.i., average viral titers (n = 3) were determined by a microtiter plaque assay. HFFs were inoculated for 2 h with serially diluted lysed cultures. Plaque numbers were determined 11 d p.i. by fluorescence microscopy. At 3 d p.i., all IFN treatments significantly reduced viral titers as compared to vehicle-treated cultures (P < 0.001, one-way ANOVA and Tukey's post hoc *t *test). At 4 d p.i., only cells treated with GCV or combination IFN treatments inhibited viral titers as compared to vehicle-treated HFFs (P < 0.001, one-way ANOVA and Tukey's post hoc *t *test). Significant reduction denoted by a single asterisk. Inset: Represents HCMV titers determined over 11 d for (◆) vehicle-treated and (○) IFN-β and IFN-γ-treated HFFs. The dashed line represents the lower limit of detection of the plaque assay (20 PFU/ml) used to measure viral titers.

To further characterize the inhibitory effect of type I IFNs (IFN-α or IFN-β) and type II IFN (IFN-γ) treatment, four-day viral growth assays were performed. In cultures treated with IFN-α, IFN-β or IFN-γ, viral replication was undetectable or below the lower limit of detection at 1 and 2 days (d) post-infection (p.i.). At 3 d p.i., however, HCMV replicated to average titers of 8350, 1050 or 985 PFU/ml in IFN-α-, IFN-β- or IFN-γ-treated cultures, respectively (Figure 3). While vehicle-treated cells replicated to average titers of 3.2 × 10^4 ^PFU/ml, viral titers recovered from cells treated with IFNs separately were reduced by 6-, 23- or 25-fold, respectively. Moreover, at 4 d p.i., viral titers in cells treated with IFNs separately were equal to viral titers recovered from vehicle-treated cultures. Consistent with our plaque reduction assays, we observed a similar enhanced inhibitory effect when HFFs were treated with a combination of type I and type II IFNs. In cultures treated with 100 IU/ml each of IFN-α and IFN-γ or IFN-β and IFN-γ, HCMV replication was detectable beginning at 3 d p.i. yielding titers at or below the lower limit of detection of the assay. Compared to HCMV titers of 1 × 10^5 ^PFU/ml at 4 d p.i. in vehicle-treated HFFs, treatment with IFN-α and IFN-γ or IFN-β and IFN-γ inhibited HCMV replication in HFFs by an average of 3125- or 5000-fold, respectively. When compared to ganciclovir (GCV)-treated cells, a known DNA synthesis inhibitor of HCMV, the level of inhibition achieved in GCV-treated cultures was comparable to that in IFN-α and IFN-γ- or IFN-β and IFN-γ-treated cultures at 3 and 4 d p.i. (Figure 3). In addition, the potent inhibitory effect observed in the presence of IFN-β and IFN-γ was maintained up to 11 d p.i. (Figure 3, inset), indicating that the effect was not merely a delay in viral replication.

### Treatment with IFN-α/β and IFN-γ does not prevent HCMV entry into HFFs

**Figure 4:**
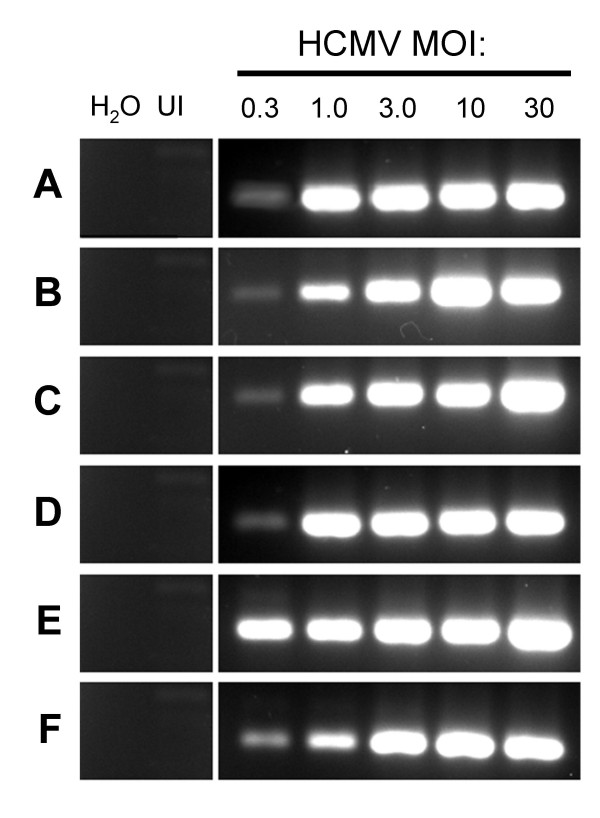
Inhibition of HCMV by IFN-α, IFN-β and/or IFN-γ is not a result of decreased viral entry into cells. Ethidium bromide-stained IE exon 4 PCR products amplified from HCMV-infected HFFs pre-treated with either vehicle (A) or 100 IU/ml of IFN-α (B), IFN-β (C), IFN-γ (D), IFN-α and IFN-γ (E) or IFN-β and IFN-γ (F). From left to right, PCR products were amplified from H_2_O control, 100 ng of uninfected (UI) HFF DNA or 100 ng of HCMV-infected HFF DNA harvested from cells inoculated for 2 h at MOIs of 0.3 to 30. GAPDH PCR products were run along side IE exon 4 PCR products and served as internal loading controls (data not shown).

The HCMV replication cycle is a multistep process, beginning with viral attachment and entry into the host target cell [2]. To investigate the mechanism(s) by which IFN-α/β and IFN-γ synergistically inhibit HCMV replication, we first examined the effect of IFNs on HCMV entry into HFFs. Cells were treated with vehicle or IFNs for 12 hours (h) prior to infection with HCMV. Two h after viral adsorption, DNA was isolated from the HCMV-infected cells and PCR was used to amplify a 373 bp fragment of the HCMV IE gene (Figure 4). For each treatment group, the PCR product yield increased as a function of viral multiplicity of infection (MOI). At all MOIs tested, the amount of PCR product amplified from HFFs treated with IFNs (Figure 4B–F) was comparable to that of vehicle-treated HFFs (Figure 4A). Co-amplification of a GAPDH 239 bp PCR product served as an internal loading control for normalization of PCR product between treatment groups (data not shown). The amplification of similar levels of PCR products from HFFs suggests that the synergistic inhibitory effect of IFN-α/β and IFN-γ does not occur at the level of viral entry.

### IFN-α/β and IFN-γ inhibit HCMV IE mRNA expression

**Figure 5:**
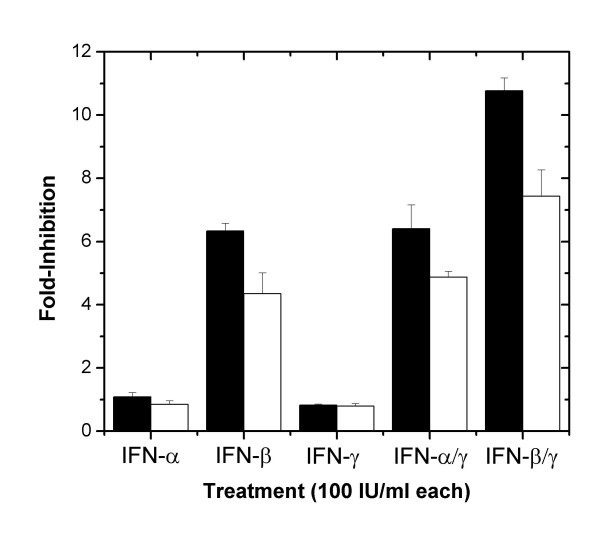
IFN-α, IFN-β and/or IFN-γ inhibit HCMV IE mRNA expression. SYBR green real-time PCR analyses of IE1 and IE2 mRNA expression in vehicle- or IFN-treated HFFs 6 h p.i. (n = 3). Presented are fold-inhibition ± standard deviation in IE1 (■) and IE2 (□) mRNA expression in each treatment group. Differences in gene expression were determined as described in Methods.

HCMV gene expression is temporally regulated in that the IE genes (IE1 and IE2) are the first class of viral genes expressed after HCMV entry into the cell [44]. Although limited studies have examined the effect of IFN-β or IFN-γ treatment on HCMV IE mRNA expression, the conclusions of these studies are conflicting, most likely due to differences in both IFN and cell type [45,46]. To assess the effect of IFN treatment on IE gene expression, real-time PCR analyses of IE1 and IE2 mRNA levels in IFN-treated cells were performed. Figure 5 summarizes the fold-repression in IE1 and IE2 mRNA levels in IFN-treated cultures as compared to vehicle-treated controls. At 6 h p.i., IE mRNA levels in HFFs treated individually with either IFN-α or IFN-γ were inhibited by < 2-fold, whereas in cells treated with both IFN-α and IFN-γ, IE1 or IE2 mRNA expression was inhibited by 6- or 5-fold, respectively. A more enhanced inhibitory effect was observed in HFFs treated with both IFN-β and IFN-γ. In these cultures, IE1 or IE2 mRNA expression was repressed by 11- or 8-fold, respectively. Interestingly, the degree of IE mRNA inhibition observed in HFFs treated with IFN-β alone was greater than that observed in cultures treated with IFN-α alone, suggesting that type I IFN-mediated inhibition of IE mRNA expression is better facilitated by treatment with IFN-β rather than IFN-α.

### IFN-α/β and IFN-γ inhibit HCMV IE protein expression

**Figure 6:**
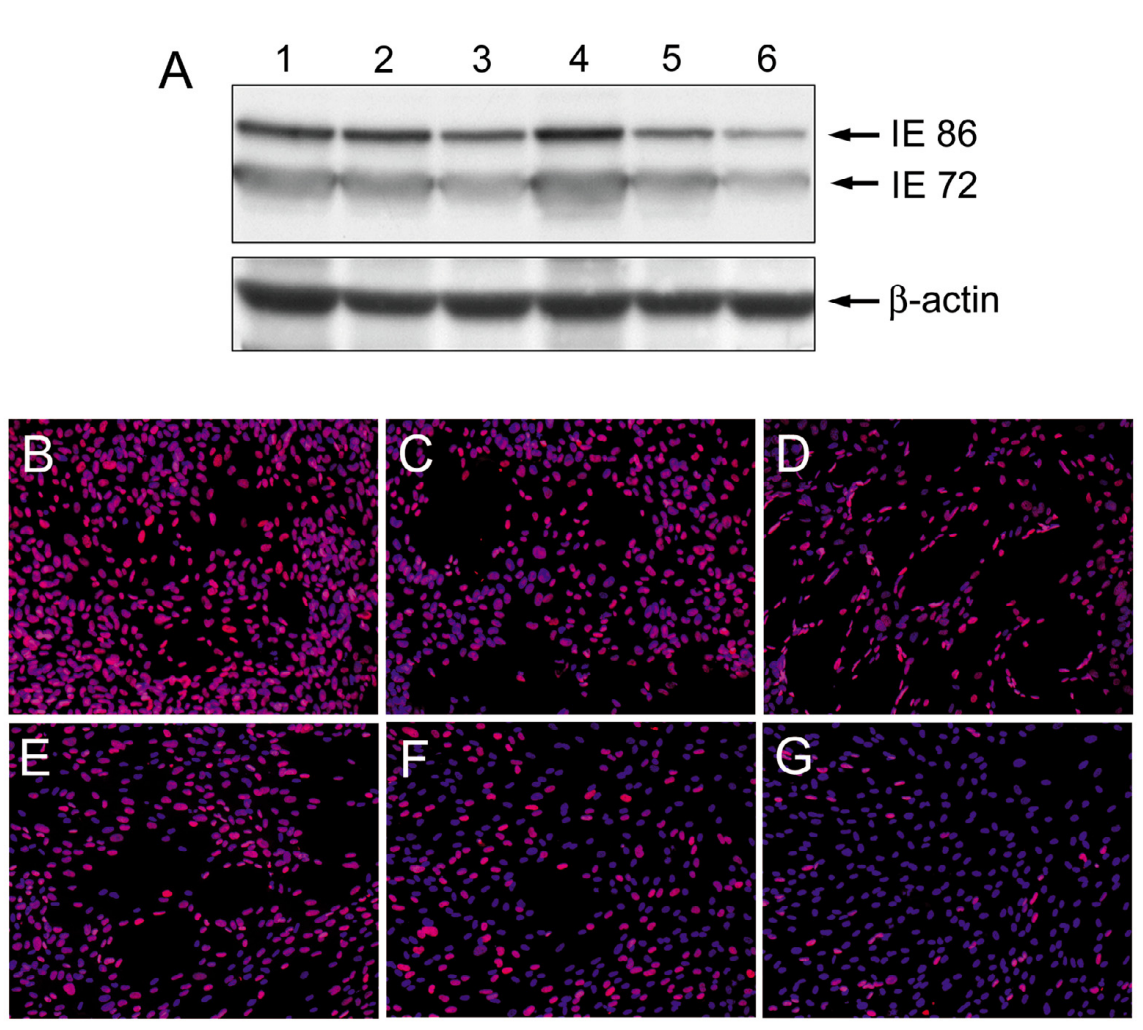
IFN-α, IFN-β and/or IFN-γ inhibit HCMV IE protein expression. (A) HFFs were pre-treated with either vehicle (1) or 100 IU/ml of IFN-α (2), IFN-β (3), IFN-γ (4), IFN-α and IFN-γ (5) or IFN-β and IFN-γ (6) 12 h prior to infection with HCMV. At 12 h p.i., cells were harvested and equal amounts of total protein were examined for IE protein (IE72, IE86) expression by western blot analyses. (B-G) Vehicle- or IFN-treated cells were infected with HCMV and the nuclear proteins IE72/86 were detected by indirect immunofluorescence 5 d p.i. Representative images (100X) from cultures treated with (B) vehicle, (C) IFN-α, (D) IFN-β, (E) IFN-γ, (F) IFN-α and IFN-γ or (G) IFN-β and IFN-γ. Immunofluorescent labeling: HCMV IE72/86 – Alexa Fluor 568 (red), nucleus – DAPI (blue), overlaid (pink).

IE protein expression plays a pivotal role in controlling subsequent viral and cellular gene expression during productive HCMV infection [47], such that an inhibitory effect at this level would significantly impair viral replication. To determine whether the inhibitory block in IE mRNA expression correlated with decreased IE protein expression in IFN-treated cultures, western blot analyses were performed (Figure 6A). At 12 h p.i., a slight reduction in IE72 and IE86 protein expression was observed in HFFs treated with IFN-β, but not with IFN-α or IFN-γ. Moreover, IE72 and IE86 protein expression was decreased in cells treated with both type I and type II IFNs, with the greatest inhibitory effect observed in HFFs treated with both IFN-β and IFN-γ. This inhibitory block in IE protein expression was consistent throughout a 48 h time period (data not shown).

If IFN-α/β and IFN-γ synergistically inhibit HCMV replication through inhibition of IE gene expression, we hypothesized that this inhibitory effect would be maintained after multiple rounds of viral replication. To address this question, IE protein expression was analyzed by indirect immunofluorescence over a 5-day period. For all treatment groups, IE protein expression was detected as early as 1 h p.i.; however, as viral replication progressed IE protein expression among IFN-treated groups varied (data not shown). Notably, by day 5 p.i., nearly 100% of the cells treated with vehicle, IFN-α or IFN-β alone stained positive for IE72/86, and approximately 87% of the cells treated with IFN-γ alone were expressing the IE proteins (Figure 6B–6E). In contrast, the percentage of cells expressing IE proteins was significantly reduced (P < 0.001) in the treatment groups that received combination IFNs, with only 46% of IFN-α and IFN-γ-treated HFFs and 21% of IFN-β and IFN-γ-treated HFFs positive for IE72/86 (Figure 6F, 6G). The observed differences suggest that in cells treated with both type I and type II IFNs, IE expression is (1) differentially regulated and/or (2) viral spread is severely hindered.

## Discussion

The immune response to viral infection is responsible for preventing viral dissemination and uncontrolled replication within the host. Following viral infection, type I IFNs are secreted by infected cells and function to induce an antiviral state in neighboring uninfected cells. Infiltrating immune cells, such as NK cells and macrophages, secrete numerous chemokines and cytokines that contribute to the overall antiviral response. Upon activation of the adaptive immune response, T-cells can further add to the milieu of immune cytokines present at the site of viral infection by secreting additional cytokines, including IFN-γ. Although several studies have examined the effects of proinflammatory cytokines on HCMV replication *in vitro*, these studies are limited as they only examine the effect of one type of cytokine on viral replication rather than examining cytokines in combination. In support of the latter, recent studies have shown that type I and type II IFNs function, in synergy, to inhibit both RNA and DNA viruses, including HCV [41], SARS-CoV [39], Lassa virus [40] and HSV-1 [20]. These studies may more accurately represent the *in vivo *inflammatory response that results after viral infection. The results presented herein are consistent with this hypothesis and establish that type I (IFN-α and IFN-β) and type II (IFN-γ) IFNs synergistically inhibit the replication of HCMV.

In the present study we have demonstrated that combination treatment with type I and type II IFNs renders cells non-permissive to HCMV replication *in vitro*. The inhibitory effect by IFN-α/β and IFN-γ was synergistic in nature (Table 2, Figure 2C, 2D) and the degree of inhibition was not matched by increasing the concentrations of each individual IFN (Table 1, Figure 2A). These results indicate that the observed IFN-induced antiviral effects are a direct result of the presence of two distinct types of IFNs. Moreover, inhibition of HCMV replication in cells treated with IFN-α/β and IFN-γ was observed in both HFF and embryonic lung fibroblasts (MRC5) (data not shown) infected with either Towne-GFP (see Methods) or another laboratory strain, AD169 (data not shown). The mechanism(s) by which HCMV replication is inhibited remains unclear. Type I and type II IFNs may synergize by acting on one or more different stages of the HCMV lytic cycle such as (1) viral attachment, (2) viral entry, (3) IE gene expression, (4) early gene expression, (5) DNA replication, (6) late gene expression, (7) virus assembly or (8) viral egress and maturation. To address the question of attachment and entry, PCR was used to amplify viral DNA from IFN-treated and vehicle-treated cultures shortly after infection. As previously observed [20,46], IFN treatment did not prevent viral entry into cells as indicated by equal PCR product yield from all treatment groups (Figure 4). These data indicate that IFNs exert their inhibitory effects at a step after viral attachment and entry.

Previously, Yamamoto, *et al. *(1987) demonstrated that treatment of cells with both IFN-α and IFN-γ potently inhibits HCMV replication; however, this study neither determined whether the effect was synergistic nor identified the mechanism of inhibition. However, the authors suggested that IFN-mediated inhibition of HCMV might occur at or prior to early gene expression [48]. Similarly, over the course of our experiments utilizing the Towne-GFP strain, it was noticed that very few cells expressed green fluorescent protein (GFP) when treated with IFN-α/β and IFN-γ together (data not shown). In this recombinant Towne strain, GFP expression is driven by the early promoter UL127. The lack of GFP-positive cells in IFN-α/β and IFN-γ-treated groups suggested to us that the synergistic antiviral activities mediated by type I and type II IFNs occurred at a stage prior to early gene expression. Previous, studies have shown that type I or type II IFN treatment can inhibit HCMV IE mRNA expression [46] and/or HCMV IE protein expression [45,46]. Using real-time PCR, we showed that while IFN-α, IFN-β or IFN-γ treatment inhibited IE mRNA expression by 2–6 fold at 6 h p.i., combination IFN-α and IFN-γ or IFN-β and IFN-γ treatment inhibited IE mRNA expression by 6–11 fold. Of note, this inhibitory effect was abolished by 24 h p.i. (data not shown), suggesting that IE mRNA expression is delayed by IFN treatment. The observed decrease in viral IE mRNA expression was accompanied by a decrease in IE protein expression, as viral IE protein expression was reduced in HFFs treated with both type I and type II IFNs (Figure 6A). Furthermore, immunofluorescent microscopy of IE protein expression revealed that nearly 100% of vehicle- and individual IFN-treated cells expressed IE72/86 5 d p.i., as compared to 46% or 21% of cells treated with IFN-α and IFN-γ or IFN-β and IFN-γ, respectively (Figure 6B–6G). It appears that although individual IFN treatment results in a marginal inhibition in IE expression early in infection, the effect is not maintained as demonstrated by high viral titers at 4 d p.i. (Figure 3) and increased IE protein expression at 5 d p.i. (Figure 6A–6E). Additionally, HCMV cytopathic effect, characterized by enlarged cells containing intranuclear and cytoplasmic inclusions, increased over time in vehicle- and individual IFN-treated groups, while morphology was unchanged in cells treated with IFN-α/β and IFN-γ (data not shown). Collectively, these data suggest that the synergistic inhibition of HCMV replication by IFN-α/β and IFN-γ may involve, at least in part, the regulation of IE gene expression. The significance of an inhibitory block at this level is evident when the phenotype of IE1 mutant viruses is considered. Greaves and colleagues have demonstrated that HCMV IE1 mutants exhibit a diminished replication efficiency and a reduced ability to form plaques, as well as defective early gene expression [47,49,50]. Interestingly, in the presence of both type I and type II IFNs, HCMV shows similar replication and gene expression defects. Although our data suggest that IE gene regulation contributes to the synergistic inhibition of HCMV replication by IFN-α/β and IFN-γ, other mechanisms may also affect this dramatic response. Accordingly, the decrease in IE protein levels exceeds that in IE mRNA levels in response to IFN-α/β and IFN-γ, suggesting that additional regulation at the level of translation, post-translational processing and/or protein stability may be involved. Delineating the other putative regulatory mechanisms that contribute to IFN-α/β and IFN-γ synergistic inhibition of HCMV replication is the focus of ongoing studies.

Type I IFNs (IFN-α and IFN-β) and type II IFN (IFN-γ) activate distinct but related Jak/STAT signal cascades resulting in the transcription of several hundred IFN-stimulated genes [26]. Although similar genes are activated by all three IFNs, Der, *et al. *(1998) have identified numerous genes differentially regulated by IFN-α, IFN-β or IFN-γ [51]. In particular, IFN-β stimulation induces twice as many genes as compared to IFN-α. This differential regulation of IFN-induced genes may explain in part the fact that the level of inhibition observed in HFFs treated with both IFN-β and IFN-γ was consistently greater than that observed in cells treated with both IFN-α and IFN-γ, although both IFN-α and IFN-β bind to the same receptor. Similarly, when compared individually, IFN-β consistently inhibited HCMV replication and IE gene expression to levels greater than IFN-α. Therefore, to better understand the cellular factors involved in the synergistic inhibition of HCMV, the profile of IFN-stimulated genes present in cells treated with both type I and type II IFNs should be further examined.

## Conclusion

Guidotti and Chisari have reported a model of noncytolytic control of viral infections by the innate and adaptive immune response, in which cytokines are implicated as having a direct role in viral clearance [21]. Here we demonstrate that IFN-γ, together with the innate IFNs (IFN-α/β) synergistically inhibits the replication of HCMV *in vitro*. We hypothesize that IFN-γ produced by activated cells of the adaptive immune response may potentially synergize with endogenous type I IFNs to inhibit HCMV dissemination and facilitate the establishment and/or maintenance of latency in the host. Further studies are required to evaluate the role(s) of both type I and type II IFNs in the regulation of HCMV replication.

## Methods

### Cells, viruses and interferons

HFFs (Viromed, Minneapolis, MN) were maintained in minimal essential medium (MEM) supplemented with 10% fetal bovine serum, penicillin G (100 U/ml), streptomycin (100 mg/ml), 2 mM L-glutamine, 1 mM sodium pyruvate and 100 μM non-essential amino acids at 37°C in 5% CO_2_. HCMV strain RVdlMwt-GFP was propagated in HFFs as previously described [52]. RVdlMwt-GFP, referred to as Towne-GFP throughout this manuscript, is a recombinant of HCMV strain Towne that expresses GFP under the control of the early promoter UL127. This virus was kindly donated by Mark F. Stinski and has been previously described [53].

Recombinant human universal IFN-α, IFN-β and IFN-γ (PBL Biomedical Laboratories, New Brunswick, NJ) were added to cell cultures 12 h prior to HCMV infection and maintained after viral infection. Concentrations of 100 IU/ml of each IFN were used in all experiments unless stated otherwise.

### Plaque reduction and viral replication assays

For plaque reduction assays, vehicle- and IFN-treated HFFs were infected with a fixed inoculum of Towne-GFP. After 2 h adsorption, the inoculum was removed and medium containing 1.0% methylcellulose (Fisher Scientific, Houston, TX) and the respective IFN(s) was added to the cells. Plaque numbers were determined 14 d later by fluorescent microscopy (Nikon TE300 inverted epifluorescent microscope, Nikon USA, Lewisville, TX).

For viral replication assays, vehicle- and IFN-treated HFFs were infected with Towne-GFP at a MOI of 2.5. After 2 h adsorption, the inoculum was removed, monolayers were washed twice with 1X PBS, and fresh IFN-containing medium was returned to each well. For GCV-treated groups, 100 μM GCV (Sigma, St. Louis, MO) was added to culture medium immediately following infection. One, 2, 3 or 4 d p.i. cells and medium were harvested and titers of infectious virus were determined by a microtiter plaque assay on HFFs [20].

### Synergy assays

To determine the degree of antiviral interaction between type I and type II IFNs, interaction indexes were calculated using the inequalities: d_a_/D_a_+d_b_/D_b _> 1 and d_a_/D_a_+d_b_/D_b _<1, where d_a _and d_b _are the IFN concentrations needed to jointly produce the effect under consideration, and D_a _and D_b _are the IFN concentrations capable of producing the effect on their own, termed isoeffective doses [42]. Interaction index values of less than 1 indicate synergism, interaction index values greater than 1 indicate antagonism and interaction index values equal to 1 indicate additivity. Isobolograms were also generated to geometrically assess the degree of antiviral interaction between type I and type II IFNs, as previously described [43]. Using the guidelines described by Berenbaum [43], isoboles were generated for IC_95 _values at various concentrations of IFN-α or IFN-β in the presence of various concentrations of IFN-γ. Concave isoboles are indicative of synergy while convex isoboles are indicative of an antagonistic effect (Figure 2B). For all synergy experiments, HCMV plaque reduction assays were conducted as described above.

### Viral entry assay

Vehicle- and IFN-treated HFFs were inoculated with Towne-GFP at MOIs of 0.3, 1, 3, 10 or 30. After 2 h adsorption, the inoculi were removed, cells were washed twice with 1X PBS, and subsequently treated with 0.05% trypsin for 5 minutes to ensure the release of virus that had adhered but had not entered the cells. Cells were pelleted and washed twice with 1X PBS to remove trypsin and non-adhered virus. DNA was isolated from each sample by a standard phenol:chloroform DNA extraction procedure [54], and HCMV-specific oligonucleotide primers were used to amplify a 373 bp product corresponding to exon 4 of the HCMV IE gene, as described previously [55]. PCR products were resolved in a 2% agarose gel and imaged using an Alpha Innotech gel documentation system (Alpha Innotech, Corp., San Leandro, CA).

### Real-time PCR

Vehicle- and IFN-treated HFFs were infected with Towne-GFP at a MOI of 2.5. Six h p.i., total RNA was prepared using a RNeasy Mini Prep kit (Qiagen, Inc., Valencia, CA) according to the manufacturer's instructions. Samples were treated with DNase I (Ambion, Inc., Austin, TX), RNA concentration and purity were determined spectrophotometrically (A_260_/A_280_) and 250 ng was reverse transcribed in a total volume of 20 μl using the iScript cDNA Synthesis Kit (Biorad, Hercules, CA) according to the manufacturer's instructions. For real-time PCR, 1 μl of cDNA was amplified in 1X iQ SYBR Green Supermix containing specific primer pairs using the iCycler iQ Real-Time PCR Detection System (Biorad). The optimal primer concentrations and sequences were as follows: 200 nM IE1, sense 5' CAAGTGACCGAGGATTGCAA 3', antisense 5' CACCATGTCCACTCGAACCTT 3' ; 200 nM IE2, sense 5' TGACCGAGGATTGCAACGA 3', antisense 5' CGGCATGATTGACAGCCTG 3' [56]; 100 nM 18S rRNA, sense 5' GAGGGAGCCTGAGAAACGG 3', antisense 5' GTCGGGAGTGGGTAATTTGC 3'. All samples were run on the same plate where those for the internal control (18S rRNA) and those for the genes of interest were each run in triplicate, for each of 3 independent RNA preparations. PCR parameters were as follows: an initial step to denature at 95°C for 30 seconds followed by 40 cycles at 95°C for 15 seconds and anneal/extend at 60°C for 45 seconds. Following amplification, melt curves were generated to confirm the specificity of each primer pair with 80 cycles of increasing increments of 0.5°C beginning with 55°C for 30 seconds. Relative quantification of the target genes in comparison to the 18S reference gene was determined by calculating the relative expression ratio (R) of each target gene as follows: R = (E_target_^)ΔCT(vehicle-sample)^/(E_18S_^)ΔCT(vehicle-sample) ^[57]. Differences in gene expression between the IFN-treated cells and the vehicle-treated control cells were expressed as fold-inhibition.

### Western blotting

Vehicle- and IFN-treated HFFs were infected with Towne-GFP at a MOI of 2.5. Twelve h p.i., the cells were harvested in 500 μl of 1X RIPA buffer containing a protease inhibitor cocktail (Roche Applied Science, Indianapolis, IN) and 1 mM PMSF. Lysates were sheared 3X with a 27G 1/2 needle and cell debris was pelleted by centrifugation at 14,000 r.p.m. at 4°C. Total protein concentrations from cleared supernatants were estimated with a Micro BCA™ Protein Assay Kit (Pierce, Rockford, IL), 50 μg of total protein were resolved on 10% SDS-polyacrylamide gels and transferred by blotting to PVDF membranes (Amersham Biosciences, Piscataway, NJ). Non-specific reactivity was blocked with 5% nonfat dried milk in Tris-buffered saline containing 0.1% Tween-20 (TBST) for 1 h at room temperature and blots were incubated for 1 h at room temperature with a polyclonal antibody that recognizes the HCMV IE proteins (IE72/86), kindly provided by Daniel N. Streblow [58]. The blots were then washed in TBST and incubated with donkey anti-rabbit IgG conjugated to horseradish peroxidase (1:5000; Amersham Biosciences) for 1 h at room temperature. Antigen-antibody complexes were detected using an enhanced chemiluminescence system (Amersham Biosciences). Blots were subsequently washed in TBST and tested for immunoreactivity to a rabbit polyclonal antibody to human β-actin (Sigma; loading control).

### Indirect immunofluorescence

Vehicle- and IFN-treated HFFs were infected with Towne-GFP at a MOI of 1.0. Five d p.i., cells were washed 3X with 1X PBS, fixed with 1:1 methanol/acetone for 10 minutes at room temperature, washed again with 1X PBS, and blocked with 4% BSA/PBS for 15 minutes at room temperature. Cells were incubated for 1 h at 37°C with a HCMV IE antibody (IE72/86 kD; Chemicon #MAB810, Temecula, CA) diluted 1:200 in 0.5% BSA/PBS. Cells were then stained with 1:50 Alexa Fluor 568-conjugated goat anti-mouse IgG F(ab')_2 _(Molecular Probes, Eugene, OR) for 30 minutes at 37°C, followed by a 2 minute incubation with 1 μM 4',6-diamidino-2-phenylindole, dihydrochloride (DAPI; Molecular Probes) at room temperature. Cells were coverslipped and mounted in Prolong Antifade mounting medium (Molecular Probes), visualized on a Zeiss Axio Plan II microscope (Thornwood, NY) and images were analyzed with deconvolution SlideBook™ 4.0 Intelligent Imaging software (Intelligent Imaging Innovations, Denver, CO). To determine the number of HCMV-infected cells, three fields of view (100X) for each treatment group were considered and the percent of IE-positive cells was calculated as: (average number of IE-stained cells/average number of DAPI-stained cells)×100.

### Statistics

Data are presented as the means ± standard error of the means (sem). Data from IFN-treated groups were compared to vehicle-treated groups and significant differences were determined by one-way analysis of variance (ANOVA) followed by Tukey's post hoc *t *test (GraphPad Prism^© ^Home, San Diego, CA).

## Competing interests

The author(s) declare that they have no competing interests.

## Authors' contributions

BS and HL conceived of the study, participated in the experimental design, performed all experiments and drafted the manuscript. RG and CM participated in the coordination and design of the study. All authors read and approved the final manuscript.
